# Capillaroscopic appearance of nailfold vasculature of diabetic nephropathy patients

**DOI:** 10.20945/2359-3997000000475

**Published:** 2022-05-12

**Authors:** Ustun Yilmaz, Ayse Ayan, Seyit Uyar, Ayca Inci, Hakan Ozer, Fikriye Tuter Yilmaz, Gulay Demirtas, Mehmet Kok, Abdullah Tokuc

**Affiliations:** 1 SBU Antalya Training and Research Hospital Department of Nephrology Antalya Turkey Department of Nephrology, SBU Antalya Training and Research Hospital, Antalya, Turkey; 2 SBU Antalya Training and Research Hospital Department of Rheumatology Antalya Turkey Department of Rheumatology, SBU Antalya Training and Research Hospital, Antalya, Turkey; 3 SBU Antalya Training and Research Hospital Department of Internal Medicine Antalya Turkey Department of Internal Medicine, SBU Antalya Training and Research Hospital, Antalya, Turkey; 4 SBU Antalya Training and Research Hospital Department of Neurology Antalya Turkey Department of Neurology, SBU Antalya Training and Research Hospital, Antalya, Turkey

**Keywords:** Diabetic nephropathy, nailfold videocapillaroscopy, type 2 diabetes

## Abstract

**Objectives:**

Diabetic nephropathy is a microvascular complication of diabetes and the most common cause of end-stage renal failure throughout the world. Videocapillaroscopy is a simple and noninvasive method that can display capillaries in the nail bed at the micron level. A few studies have been conducted on detecting retinopathy, another important diabetic microvascular complication, with videocapillaroscopy; however, no comprehensive study has been performed on diabetic nephropathy. We aimed to determine the relationship between nephropathy and capillaroscopic changes.

**Subjects and methods:**

Capillaroscopic findings of 144 patients with type 2 diabetes and 88 healthy controls were assessed prospectively by nailfold videocapillaroscopy. Twelve capillaroscopic findings were evaluated in all subjects.

**Results:**

Patients with albuminuria had more capillary aneurysms (15.5%), more microhemorrhages (15.5%), greater tortuosity (76.3%), more neoformations (29.9%), more bizarre capillaries (49.5%) and more bushy capillaries (20.6%) than the control group. In logistic regression analysis, tortuosity was significantly correlated with albuminuria (OR: 2.451, p = 0.048).

**Conclusions:**

Our findings show that the application of nailfold videocapillaroscopy can detect microvascular abnormalities in the nail bed that occur in diabetes mellitus patients compared to healthy people. Although there was no difference in the microvascular changes among the stages of diabetic nephropathy, a relationship between tortuosity and albuminuria was identified by logistic regression analysis. Nailfold videocapillaroscopy may be a new application that can be used to screen the microvascular changes that occur in diabetes mellitus.

## INTRODUCTION

**Figure 1:**
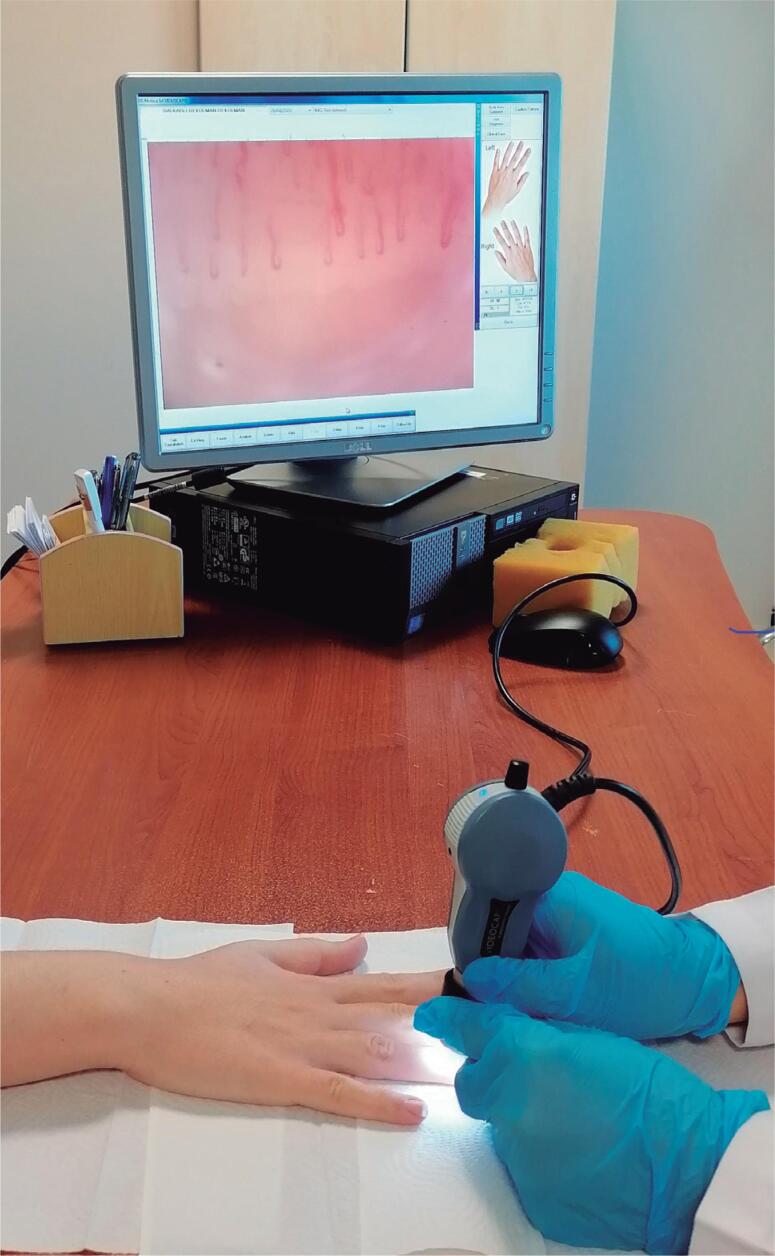
Nailfold videocapillaroscopy application method.

Nailfold videocapillaroscopy (NVC) is a simple, noninvasive, reliable diagnostic method that is used in the examination of nailfold dermal capillaries (Figure 1). It is an important method for the evaluation of patients with Raynaud's phenomenon. Microvascular findings in scleroderma, mixed connective tissue disease and dermatomyositis can also be easily determined with NVC (1,2). Microvascular vessels are affected not only in rheumatological diseases but also in several systemic diseases, such as diabetes mellitus (DM).

DM is a chronic, life-long, metabolic disease, and because of microvascular, macrovascular, acute and chronic complications, it significantly reduces quality of life and cause morbidity, mortality and an economic burden to society. Diabetic vascular complications are the most common cause of diabetes-related morbidity and mortality (3). The majority of diabetic complications occur because of angiopathy and endothelial dysfunction. As a result of diabetic vascular complications, neurological, cardiac and renal pathologies and effects on peripheral vasculature can occur. Diabetic nephropathy (DN) is one of the microvascular complications of DM and is the most common cause of end-stage renal failure throughout the world. An increase in the levels of advanced glycosylation end-products, inflammatory cytokines, oxidative stress, growth factors and intracellular signal molecules, which occurs in the pathogenesis of DN, leads to a deterioration in the vascular endothelial structure. By leading to these impairments in the endothelial structure, factors that play a role in pathogenesis can cause capillary damage. Consequently, the predominant structural changes are mesangial expansion, thickening of the glomerular basal membrane, podocyte injury and glomerular sclerosis (4–6).

Several studies have used NVC to evaluate microvascular areas in systemic diseases such as DM, and significant findings were observed in these studies (7–11). NVC was determined to be an effective method in the early diagnosis and determination of diabetic complications in these studies (9–11). However, to our knowledge, no research has been conducted that investigates the relationship between DN and NVC in detail. In this study, we aimed to evaluate nailfold capillaries in type 2 diabetes mellitus (T2DM) patients to determine any correlation between the development of nephropathy and changes in nailfold capillaries.

## SUBJECTS AND METHODS

### Patients

The study included 144 patients followed up for T2DM in the Departments of Nephrology and Internal Medicine of the University of Health Sciences Antalya Training and Research Hospital between January 2018 and November 2018 and a control group of 88 healthy individuals who presented at the Department of Internal Medicine.

T2DM patients were separated into groups of patients in the normoalbuminuric, microalbuminuric and macroalbuminuric stages according to the albumin/creatinine ratio (ACR) as measured with a spot urine test. Patients with blood pressure values above 140/90 mmHg were considered to have hypertension, and patients with LDL levels >100 mg/dL and triglyceride levels >150 mg/dL were considered to have hyperlipidemia. The exclusion criteria were a history of Raynaud's phenomenon and collagen tissue disease, use of drugs that could affect fibrinolysis metabolism (e.g., glucocorticoids and oral contraceptives), smoking, alcohol consumption and occupational risk of nailfold microtrauma (e.g., farmers, gardeners). Patients who were under 18 years old and whose glomerular filtration rate (GFR) was lower than 60 mL/min were also excluded. All of the patients were evaluated by a nephrologist for DN and by a rheumatologist for capillaroscopic assessment.

Approval for the study was granted by the local ethics committee. All study participants provided written informed consent for participation in the study.

### Nephrological evaluation

DN and its stages were evaluated by albumin excreted in the urine. For the screening of albumin in the urine, the ACR was examined in the first morning urine. DN stages were defined according to the American Diabetic Association (ADA) criteria as 1) normoalbuminuric stage = no albumin expressed in the urine (<30 mg/day), 2) microalbuminuric stage = 30-299 mg/day albumin expressed in the urine, and 3) macroalbuminuric stage = ≥300 mg/day albumin expressed in the urine. GFR was evaluated using the CKD-EPI (Chronic kidney disease epidemiology collaboration) formula.

### Capillaroscopic evaluation

**Figure 2:**
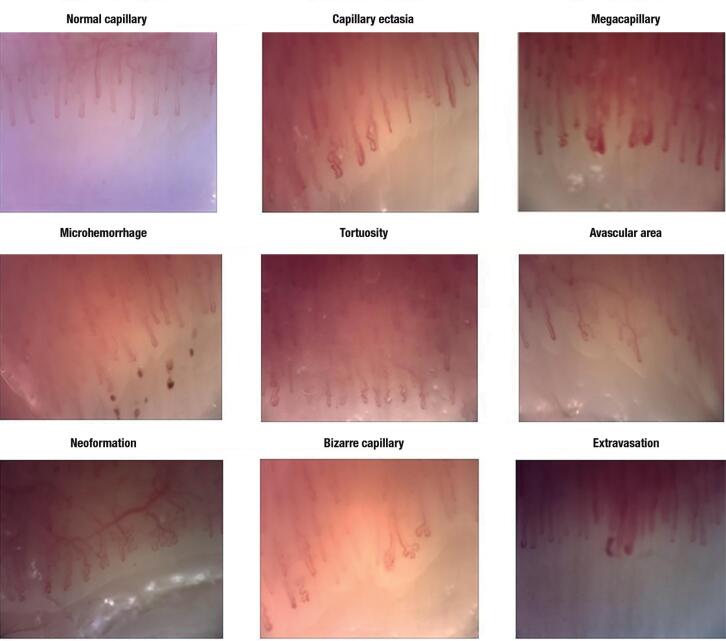
Normal and pathological videocapillaroscopy findings.

All the study participants were instructed to not drink coffee for 12 hours prior to the appointment. The patients were instructed not to remove their cuticles in the weeks prior to the examination. After resting for 15-20 minutes in a room at 22-24 °C, immersion oil was dropped onto the nailfold to obtain a clear image. The capillaroscopic examination was performed using a magnifying probe on the nailfold and transferring images at 200x magnification to a computer screen with analysis software (Videocap, DS MediGroup, Milan, Italy). The images of all 8 fingers, excluding the thumbs, were taken by a rheumatologist who was educated and blinded regarding whether the individual was a patient or a healthy control, and the images were recorded. Normal nailfold capillaries in healthy individuals are parallel to the nail surface and have the appearance of a red hairgrip at all lengths. Abnormal capillaroscopic findings were defined as follows: 1) microhemorrhage: 2 or more punctate bleeding around a single capillary in at least 2 fingers; 2) neoangiogenesis: tortuous, bush-like capillaries with marked heterogeneity in size; a) as the presence of extremely tortuous, bushy, branching, ramified and coiled capillaries; b) ≥4 capillaries within a single dermal papilla; c) thin and branching interconnected capillaries originating from a single loop; 3) tortuosity: 2 or more cross capillaries over each 1 mm length; 4) bizarre capillaries: capillaries with abnormal appearance but not resembling other defined abnormal capillaries; 5) extravasation: leakage of capillary content; 6) ectatic capillaries: capillary wall diameters between 0.02 and 0.05 μm (regular or irregular); 7) megacapillary: capillary wall diameter > 0.05 μm; and 8) avascular area: loss of at least 2 consecutive capillaries or ≤ 6 capillaries over 1 mm in length (Figure 2) (12).

### Statistical analysis

Statistical analysis was performed using IBM SPSS Statistics for Windows, Version 22.0 (IBM Corp., Armonk, NY). Fisher's exact test and Pearson chi-square analysis were performed for categorical variables. The normality assumptions were controlled by the Shapiro-Wilk test. The differences between two groups were evaluated with the Mann-Whitney U test for nonnormally distributed data. One-way ANOVA was used for comparison of parametric variables between groups, and Tukey's HSD test was used as a post hoc test for significant cases. Receiver operating characteristic (ROC) curve analysis was applied to evaluate the predictive performance of capillaroscopic findings for albuminuria detection, and the area under the curve (AUC), sensitivity and specificity were calculated and reported with 95% confidence intervals. Multivariate logistic regression analysis was performed to determine the association between capillaroscopic findings and albuminuria. Data are expressed as n (%), mean ± standard deviation (SD) or median (min-max), as appropriate. P values < 0.05 were considered statistically significant.

## RESULTS

### Characteristic features

The normoalbuminuric group (n = 47) comprised 32 females and 15 males with a mean age of 59.2 ± 10 years. The albuminuric group (n = 97) comprised 55 females and 42 males with a mean age of 61.7 ± 10.2 years. The control group (n = 88) comprised 44 females and 44 males with a mean age of 58.9 ± 7.2 years. There was no statistically significant difference between the three groups in terms of age (p = 0.114). Hypertension and hyperlipidemia history and medication were not significantly different in the normoalbuminuric and albuminuric groups (p = 0.140 and p = 0.335). HbA1c values were determined to be higher in albuminuric patients (8.5%) than in normoalbuminuric patients (7.1%) (p < 0.001). The median duration of DM was determined to be 7 years and 8 years in the two groups, respectively (p = 0.208) (Table 1).

**Tabla 1:** Demographical characteristics and frequencies of capillaroscopic findings of patients with DN (normoalbuminuric and albuminuric) and healthy controls

	Normoalbuminuric patients (n: 47)	Albuminuric patients (n: 97)	Healthy controls (n: 88)	*p*
Mean age (SD)	59.2 ± 10^a^	61.7 ± 10.2^a^	58.9 ± 7.2^b^	0.114
Male sex	15 (31.9 %)	42 (43.3 %)	44 (50)	0.130
Hypertension	24 (51.1%)	62 (63.9%)	-	0.140
Hyperlipidemia	15 (31.9%)	39 (40.2%)	-	0.335
HbA1c, % (min-max)	7.1 (5.9-10.7)	8.5 (4.7-15.3)	-	***<0.001***
Diabetes duration, median (min-max)	7 (0.5-20)	8 (0.5-28)	-	0.208
Albuminuria (mg/day) (min-max)	8 (3-28)	314 (32-5420)	-	***<0.001***
Capillary ectasia	3 (6.4%)	10 (10.3%)	0 (0)	NA
Capillary aneurysm	4 (8.5%)^a^	15 (15.5%)^a^	0 (0)^b^	***0.001***
Megacapillary	2 (4.3%)	0 (0%)	0 (0)	NA
Microhemorrhage	5 (10.6%)^a^	15 (15.5%)^a^	0 (0)^b^	***0.001***
Tortuosity	28 (59.6%)^a^	74 (76.3%)^a^	6 (6.8)^b^	***<0.001***
Avascular area	1 (2.1%)	4 (4.1%)	0 (0)	NA
Neoformation	12 (25.5%)^a^	29 (29.9%)^a^	0 (0)^b^	***<0.001***
Bizarre capillary	23 (48.9%)^a^	48 (49.5%)^a^	5 (5.7)^b^	***<0.001***
Interstitial edema	0 (0%)	0 (0%)	0 (0)	-
Bushy capillary	6 (12.8%)^a^	20 (20.6%)^a^	0 (0)^b^	***<0.001***
Meander capillary	3 (6.4%)	6 (6.2%)	0 (0)	NA
Extravasation	2 (4.3%)	0 (0%)	0 (0)	NA

Data are presented with mean ± SD, median (min-max). ANOVA with Tukey HSD, Pearson Chi-square test, Mann-Whitney U test. NA: not applicable; DN: diabetic nephropathy. Different lower-case letters denote statistically significant differences.

a b Significant difference between normoalbuminuric and albuminuric patients and the control group.

### Comparisons of the capillaroscopic findings in normoalbuminuric and albuminuric diabetic patients and healthy controls

In the capillaroscopic evaluation, the following findings were observed at a lower frequency in the control group compared to the normoalbuminuric and albuminuric patient groups: capillary aneurysm [0 (0%) vs. 4 (8.5%) vs. 15 (15.5%); p = 0.001], microhemorrhage [0 (0%) vs. 5 (10.6%) vs. 15 (15.5%); p = 0.001], tortuosity [6 (6.8%) vs. 28 (59.6%) vs. 74 (76.3%); p < 0.001], neoformation [0 (0%) vs. 12 (25.5%) vs. 29 (29.9%); p < 0.001], bizarre capillary [5 (5.7%) vs. 23 (48.9%) vs. 48 (49.5%); p < 0.001], and bushy capillary [0 (0%) vs. 6 (12.8%) vs. 20 (20.6%); p < 0.001] (Table 1).

### Comparison of the capillaroscopic findings between stages of diabetic nephropathy

No statistically significant difference was observed between the normoalbuminuric, microalbuminuric and macroalbuminuric stage groups with respect to the frequency of capillaroscopic findings (Table 2).

**Tabla 2:** Comparison of capillaroscopic findings with severity of DN stages (normoalbuminuria, microalbuminuria and macroalbuminuria)

	Normoalbuminuric patients (n: 47)	Microalbuminuric patients (n: 47)	Macroalbuminuric patients (n: 50)	*p*
Capillary ectasia	3 (6.4%)	9 (19.1%)	1 (2)	NA
Capillary aneurysm	4 (8.5%)	9 (19.1%)	6 (12)	0.299
Megacapillary	2 (4.3%)	0 (0%)	0 (0)	NA
Microhemorrhage	5 (10.6%)	6 (12.8%)	9 (18)	0.557
Tortuosity	28 (59.6%)	36 (76.6%)	38 (76)	0.117
Avascular area	1 (2.1%)	2 (4.3%)	2 (4)	NA
Neoformation	12 (25.5%)	15 (31.9%)	14 (28)	0.787
Bizarre capillary	23 (48.9%)	28 (59.6%)	20 (40)	0.156
Interstitial edema	0 (0%)	0 (0%)	0 (0)	-
Bushy capillary	6 (12.8%)	10 (21.3%)	10 (20)	0.510
Meander capillary	3 (6.4%)	4 (8.5%)	2 (4)	NA
Extravasation	2 (4.3%)	0 (0%)	0 (0)	NA

Pearson Chi-square test. NA: not applicable; DN: diabetic nephropathy.

### Comparisons of the patients with significant capillaroscopic findings according to the median years of diabetes

The median number of years of diabetes was compared between the patients with and without significant capillaroscopic findings and between the normoalbuminuric and albuminuric patients. No statistically significant difference was observed between the groups in these comparisons (Table 3).

**Tabla 3:** Comparison of median diabetes duration of patients with and without significant capillaroscopic findings and comparison of median diabetes duration of patients with significant capillaroscopic findings with normoalbuminuria and albuminuria

			Median (min-max) diabetes duration		*p^x^*	*p^y^*
			Normoalbuminuric patients	Albuminuric patients		
Capillary ectasia	- +	8 (0.5-28) 6 (0.5-17)	3 (1-6)	7.5 (0.5-17)	0.262	0.204
Capillary aneurysm	- +	8 (0.5-28) 7 (2-18)	8 (3-10)	7 (2-18)	0.504	0.802
Microhemorrhage	- +	7 (0.5-20) 9.5 (1-28)	10 (3-15)	9 (1-28)	0.198	0.965
Tortuosity	- +	9 (0.5-20) 7 (0.5-28)	7.5 (0.5-18)	7 (0.5-28)	0.525	0.663
Avascular area	- +	8 (0.5-28) 7 (5-8)	5	7 (6-8)	0.555	-
Neoformation	- +	8 (0.5-28) 7 (0.5-20)	8 (2-15)	7 (0.5-20)	0.999	0.863
Bizarre capillary	- +	8 (0.5-28) 7 (0.5-26)	10 (0.5-15)	7 (0.5-26)	0.064	0.237
Bushy capillary	- +	8 (0.5-28) 7.5 (0.5-20)	8 (2-15)	7.5 (0.5-20)	0.971	0.927
Meander capillary	- +	8 (0.5-28) 7 (0.5-12)	9 (7-11)	4.5 (0.5-12)	0.459	0.437

Mann-Whitney U test.

*p^x^* : comparison of median diabetes duration of patients with versus without significant capillaroscopic findings;

*p^y^* : comparison of median diabetes duration of patients with significant capillaroscopic findings with albuminuria versus without albuminuria.

### Multivariate logistic regression analysis with respect to the prediction of diabetic nephropathy by significant capillaroscopic findings in normoalbuminuric and albuminuric patients

A significant relationship was observed between tortuosity and albuminuria (odds ratio 2.451, confidence interval 1.003-6.005, p = 0.048) (Table 4).

**Tabla 4:** Multivariate logistic regression analysis of significant capillaroscopic findings in normoalbuminuric and albuminuric patients

	*p* value	OR (95% CI)
Capillary aneurysm	0.272	2.012 (0.578-7.005)
Microhemorrhage	0.479	1.504 (0.485-4.657)
Tortuosity	***0.048***	2.451 (1.003-6.005)
Neoformation	0.353	0.573 (0.177-1.858)
Bizarre capillary	0.292	0.643 (0.282-1.463)
Bushy capillary	0.198	2.467 (0.623-9.761)

OR: odds ratio.

### Diagnostic test predictions of significant capillaroscopic findings with respect to the determination of diabetic nephropathy

**Figure 3:**
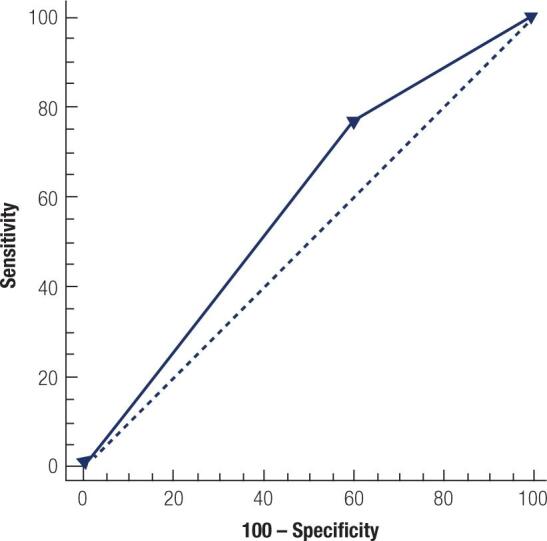
ROC curve of tortuosity.

The area under the curve (AUC) value for tortuosity was determined to be 0.584, with a sensitivity of 76.3% and specificity of 40.4% in the receiver operating characteristic (ROC) analysis (Table 5, Figure 3).

**Tabla 5:** Prediction of DN by diagnostic tests for significant capillaroscopic findings

	AUC (95% CI)	*p*	Sensitivity (95% CI)	Specificity (95% CI)
Capillary aneurysm	0.535 (0.450-0.618)	0.208	15.5 (8.9-24.2)	91.49 (79.6-97.6)
Microhemorrhage	0.524 (0.439-0.608)	0.410	15.5 (8.9-24.2)	89.4 (76.9-96.5)
Tortuosity	0.584 (0.499-0.665)	***0.048***	76.3 (66.6-84.3)	40.4 (26.4-55.7)
Neoformation	0.522 (0.437-0.606)	0.583	29.9 (21-40)	74.5 (59.7-86.1)
Bizarre capillary	0.503 (0.418-0.587)	0.951	49.5 (39.2-59.8)	51.1 (36.1-65.9)
Bushy capillary	0.539 (0.454-0.623)	0.222	20.6 (13.1-30)	87.2 (74.3-95.2)

AUC: area under curve; CI: confidence interval; DN: diabetic nephropathy.

## DISCUSSION

Microcirculation is affected in DM patients, and permanent diabetic complications can result from microvascular abnormalities. Among diabetic complications, DN is a major health concern because it results in end-stage renal failure. Early diagnosis and treatment are therefore important. Many different mechanisms play a role in the pathogenesis of DN, and microvascular damage is one important such mechanism (4–6). Due to the importance of vascular involvement in DM, NVC, which evaluates the microvascular area noninvasively, has become a research topic with respect to the early diagnosis of diabetic complications. The proportion of capillaroscopic results was shown to be slightly higher in groups of patients with diabetic retinopathy compared with groups of patients without retinopathy (9,10). In another study by Hsu and cols., correlations between the NVC score and the diabetic retinopathy severity classification, as well as between the stages of DN and the diabetic neuropathy examination score, were examined. The authors observed a strong positive association between the NVC score and the diabetic neuropathy test score alone, and no other associations were established (11). In another study with type 1 DM patients by Kuryliszyn-Moskal and cols., the capillaroscopic score was found to be significantly higher in the groups with retinopathy, nephropathy, and neuropathy as a result of comparisons made between NVC assessment of patients with and without microvascular complications (13).

Capillary aneurysm, microhemorrhage, tortuosity, neoformation, bizarre capillary and bushy capillary were observed to be higher in the DM patients than in control subjects in this study. Our findings demonstrate that NVC can detect microvascular abnormalities in DM, and this result was consistent with the literature that some capillaroscopic findings were observed at a higher rate in DM patients than in healthy controls (7,14,15). However, there was no difference among DN stage groups in double (normoalbuminuric patients versus albuminuric patients) and triple (normoalbuminuric patients versus microalbuminuric patients versus macroalbuminuric) comparisons. While studies have shown a relationship between the stages of diabetic retinopathy and NVC findings (9), no studies have been performed with the stages of DN, and our study is the first detailed study on this subject.

No statistically significant difference was observed in the NVC findings at different DN stages in the current study, and this suggests that different mechanisms (an increase in the levels of advanced glycosylation end-products, inflammatory cytokines, oxidative stress, growth factors, intracellular signal molecules and mesangial expansion, thickening of the glomerular basal membrane, podocyte injury) other than microvascular damage in DN become more prominent as DN progresses, or these phenomena may have more effects on nephropathy than microvascular damage. Prospective and new studies with more patients are needed to elucidate this issue.

In studies by Meyer and cols. and Uyar and cols., a positive correlation was observed between significant capillaroscopic findings and disease duration (9,15). In the current study, the median years of diabetes were compared between diabetic patients with and without significant capillaroscopic findings and between normoalbuminuric and albuminuric patients. No significant difference was observed between the groups in these two comparisons.

Tortuosity has been found to be more pronounced than other findings in many studies conducted on DM and capillaroscopic findings (7–9,15). Consistent with these findings in the literature, despite its low sensitivity (76.3%), tortuosity was the most valuable finding identified as a result of logistic regression analysis in this study.

This is the first comprehensive study in the literature to investigate the relationship between DN and NVC findings, and more NVC findings were examined compared to similar studies. However, the small number of patients, lack of evaluation of other diabetic complications and lack of use of the quantitative NVC score were limitations of our study.

In conclusion, the current study revealed that application of NVC can detect microvascular abnormalities in the nail bed that occur in diabetes mellitus patients. There was no difference between the stages of diabetic nephropathy in terms of microvascular changes. In more detailed analyses, tortuosity has been the prominent finding. The noninvasive nature and easy application of this technique makes it an ideal tool for predicting diabetic complications and for follow-up. As with other diabetic microvascular complications, further prospective studies may better define the clinical significance of NVC findings in DN.
